# Associations of High-Density Lipoprotein Functionality with Coronary Plaque Characteristics in Diabetic Patients with Coronary Artery Disease: Integrated Backscatter Intravascular Ultrasound Analysis

**DOI:** 10.3390/biom13091278

**Published:** 2023-08-22

**Authors:** Kohei Takata, Satoshi Imaizumi, Atsushi Iwata, Bo Zhang, Emi Kawachi, Shin-ichiro Miura, Masahiro Ogawa

**Affiliations:** 1 Department of Cardiology, Fukuoka University School of Medicine, Fukuoka 814-0180, Japan; k.takata.jp@adm.fukuoka-u.ac.jp (K.T.); miuras@cis.fukuoka-u.ac.jp (S.-i.M.); ogawam@fukuoka-u.ac.jp (M.O.); 2 Department of Clinical Laboratory and Transfusion, Fukuoka University Hospital, Fukuoka 814-0180, Japan; 3 Department of Bioethics and Medical Ethics, Fukuoka University School of Medicine, Fukuoka 814-0180, Japan; 4 Fukuoka University Health Care Center, Fukuoka 814-0180, Japan; 5 Information Technology Center, Fukuoka University, Fukuoka 814-0180, Japan; 6 Department of Biochemistry, Fukuoka University School of Medicine, Fukuoka 814-0180, Japan

**Keywords:** antioxidant capacity, coronary artery disease, cholesterol efflux capacity, high-density lipoprotein, intravascular ultrasound

## Abstract

High-density lipoprotein (HDL) functionality has been reported to be associated with coronary artery disease (CAD). However, little is known about the impact of HDL functionality on coronary atherosclerosis. Thirty-eight type 2 diabetic patients with CAD who underwent percutaneous coronary intervention were examined. Coronary atheroma burden and plaque composition of the culprit lesions were assessed using conventional gray-scale and integrated backscatter intravascular ultrasound. HDL-mediated cholesterol efflux capacity (HDL-CEC) and HDL antioxidant capacity, estimated as HDL inflammatory index (HII), were examined. The associations between HDL functionality and coronary plaques were analyzed using multivariate data analysis, including principal components analysis and orthogonal partial least squares (OPLS) models. Percent atheroma volume was correlated with HDL-CEC (r = 0.34, *p* = 0.04) but not with HII (*p* = 0.65). The OPLS model demonstrated that the percentage lipid volume was significantly associated with HDL functionality [coefficient (95% confidence interval); HDL-CEC: −0.26 (−0.49, −0.04); HII: 0.34 (0.08, 2.60), respectively]. HII exhibited the highest variable importance in projection score, indicating the greatest contribution. HDL functionality was associated with coronary plaque composition, a key component of plaque vulnerability. Our findings highlight the potential importance of HDL functionality for coronary plaque stabilization.

## 1. Introduction

Patients with type 2 diabetes mellitus (T2DM) have higher risk of cardiovascular events compared to those without T2DM, regardless of an optimal management of atherosclerotic cardiovascular disease (ASCVD) risk factors [1]. Statin therapy is a cornerstone for preventing ASCVDs, but despite their use there is still residual risk after achieving low-density lipoprotein cholesterol (LDL-C) level goals [2]. Diabetic patients frequently exhibit mixed dyslipidemia characterized by hypertriglyceridemia and low high-density lipoprotein cholesterol (HDL-C) levels. Low HDL-C level is a strong and independent risk factor of ASCVDs [3,4], and it remains a good predictor of cardiovascular events in patients with LDL-C levels <70 mg/dL [5]. While these low levels of HDL-C predict events in patients with target LDL-C levels on statins, therapies that have increased HDL-C levels, such as the addition of niacin or cholesteryl ester transfer protein inhibitors, have not shown clinical benefit in patients with ASCVDs [6,7,8]. Furthermore, some genetic mechanisms that increase HDL-C levels have been shown to not associate with a reduction in the risk of myocardial infarction [9]. Subsequently the quality of high-density lipoprotein (HDL), rather than just its levels, has attracted significant attention as a novel target for preventing ASCVDs.

HDL promotes reverse cholesterol transport (RCT) by removing cholesterol from lipid-laden macrophages in atherosclerotic plaques [10]. This ability of HDL, which is measured using an ex vivo cell-based efflux system, indicates that HDL-mediated cholesterol efflux capacity (HDL-CEC) is associated with coronary artery disease (CAD), independent of HDL-C levels [11]. In addition to antiapoptotic, antithrombotic, and fibrinolytic activities, HDL also has antioxidant capacity and reduces increased oxidative stress. A measuring system for HDL antioxidant capacity with a cell-free environment has been developed [12]. This ability of HDL is determined as HDL inflammatory index (HII), and higher HII indicates less HDL antioxidant capacity. Significant relationships of HII with ASCVDs have been demonstrated in several clinical studies [13,14]. T2DM patients showed reduced HDL-CEC and higher HII [15,16]. Patients with acute coronary syndrome (ACS) also had higher HII than those with stable CAD or those without CAD [17]. However, the role of HDL in coronary atherosclerosis assessed using an intravascular imaging modality remains limited.

Integrated backscatter intravascular ultrasound (IB-IVUS) has been developed for quantitative tissue characterization of coronary plaques [18,19]. Coronary plaques with an increased lipid composition assessed using IB-IVUS have been reported to be a useful predictor of cardiovascular events in CAD patients [20,21]. In addition, a pathohistological study reported that coronary atheroma in T2DM patients harbored a large amount of lipidic materials, resulting in vulnerable plaques [22]. Hence, the current study sought to examine the impact of HDL functionality on coronary atherosclerotic plaques using IB-IVUS imaging in T2DM patients with CAD.

## 2. Materials and Methods

### 2.1. Study Population

This study was designed to investigate the potential of HDL functionality as a residual risk factor. Of 927 patients with CAD who underwent percutaneous coronary intervention (PCI) at Fukuoka University Hospital from December 2011 to October 2014, the following patients were excluded: patients with ACS, acute heart failure, severe infection, and recent surgery or trauma given the effects of acute inflammatory conditions on HDL properties. Patients with poor IB-IVUS image quality, contraindication to the use of antiplatelet agents, and statin-intolerant patients without any other lipid-lowering therapies were also excluded. In addition, we excluded patients without T2DM or IB-IVUS imaging data due to the aim of this study: exploring the associations between HDL functionality and plaque composition in T2DM patients. Ultimately, thirty-eight T2DM patients who underwent IB-IVUS-guided PCI were included in the current study. Medical therapy in accordance with the Japanese guidelines [23,24], a standard antiplatelet therapy and optimal lipid management, had already been commenced prior to enrollment in all patients. This study was approved by the ethics committee of Fukuoka University Hospital (IRB #15-3-12).

### 2.2. Clinical Laboratory Data Analyses

Fasting blood samples were collected just before IB-IVUS-guided PCI. Stored frozen serum and plasma samples (at −80 °C) were used in this study. Serum high-sensitivity C-reactive protein (hs-CRP) and small-dense LDL-C (sd-LDL-C) levels were measured at SRL Co., Ltd. (Tokyo, Japan). A Human Apolipoprotein Milliplex kit (Millipore, Burlington, MA, USA) was used for measuring the following apolipoproteins levels: apolipoprotein A1 (apoA1), apolipoprotein B (apoB), apolipoprotein C2 (apoC2), and apolipoprotein C3 (apoC3). Other biochemical parameters including lipids were measured using routine laboratory measurements at the Department of Laboratory Medicine of Fukuoka University Hospital. The estimated glomerular filtration rate (eGFR) was calculated based on serum creatinine, age, and gender using the formula of the Japanese Society of Nephrology [25,26].

### 2.3. Measurement of the HDL-Mediated Cholesterol Efflux Capacity

We examined HDL-CEC with an ex vivo system using J774 macrophages and apoB-depleted plasma from patients as described previously [11]. The day after radiolabeling with 2 μCi/mL, the cells were washed and incubated in Dulbecco’s Modified Eagle Medium with 8-Br-cAMP for the upregulation of ATP-binding cassette A1 transporter. Efflux medium containing apoB-depleted plasma (equivalent to 2% plasma, *v*/*v*, in the medium) was added for 4 h. For every sample, radiolabeled cholesterol counts were measured for both the cell compartment and media by using Tri-Carb 2910TR liquid scintillation analyzer (PerkinElmer, Waltham, MA, USA). The cholesterol efflux activity (%) was calculated using the following formula: 100 × [radioactivity in the medium/total radioactivity (radioactivity in medium + cells extracted with NaOH/NaCl)] − cholesterol efflux activity in plasma-free medium. Three independent measurements were run in duplicate, and the coefficient of variations (CVs) were as follows: intra-assay CV 5.0%, inter-assay CV 4.9%, respectively.

### 2.4. Measurement of HDL Inflammatory Index (HII)

The HII was determined by a cell-free assay system using 2′,7′-dichlorofluorescein-diacetate (DCFH-DA) with the modification of a previously published method using oxidized PAPC (Ox-PAPC) as the fluorescence-inducing agent [17]. This assay is based on the ability of HDL to prevent the formation of inactivate oxidized phospholipids [12]. The presence of Ox-PAPC leads to the conversion of normally non-fluorescent DCFH-DA into a fluorescent form of DCFH. DCFH-DA (Invitrogen Applied Biosystems, Inc., Carlsbad, CA, USA) was first dissolved in fresh methanol at 2.0 mg/mL and then incubated in the dark at room temperature for 20 min to release DCFH. Ox-PAPC was prepared from PAPC (Avanti Polar Lipids, Inc., Alabaster, AL, USA) as previously described [12]. A total of 25 µL of Ox-PAPC (0.2 mg/mL) and a fixed volume of apoB-depleted plasma (5 µL) from the study subjects were incubated at 37 °C with phosphate buffered saline in black, flat-bottom microtiter plates for 30 min with rotation. A total of 25 µL of DCFH solution (0.2 mg/mL) was then added to each well and incubated at 37 °C for one hour with rotation. Fluorescence at an excitation wavelength of 485 nm and an emission wavelength of 535 nm was measured after incubation using a plate reader (TriStar LB941, Berthold Technologies, Bad Wildbad, Germany). HII values were calculated using the following formula: fluorescence intensity in subject samples + Ox-PAPC/fluorescence intensity in Ox-PAPC alone. Two independent measurements were run in duplicate, and the intra-assay CV was 3.0%.

### 2.5. IVUS Procedures

**Figure 1:**
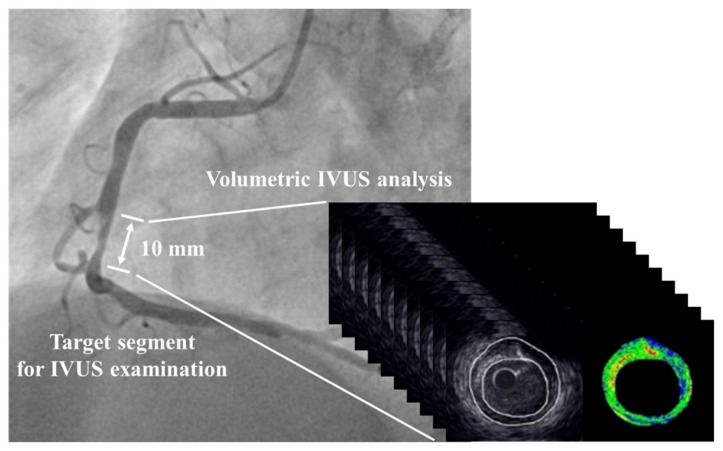
Representative Coronary Angiogram and IVUS Images. IVUS imaging was conducted just before PCI. A 10-mm target segment was defined based on the most-diseased cross section. Each cross-sectional image of IVUS and IB-IVUS spaced 1.0 mm apart. IB-IVUS = integrated backscatter intravascular ultrasound, IVUS = intravascular ultrasound, PCI = percutaneous coronary intervention.

IVUS examination was conducted for the culprit lesion of a coronary artery using an imaging catheter and a console (View IT and VISIWAVE, Terumo, Tokyo, Japan) before PCI. The IVUS catheter was advanced to the distal end of the target segment for IVUS analysis and pulled back automatically at a speed of 0.5 mm/s. To prevent coronary spasm, an optimal dose of nitroglycerin was administered into the coronary artery just before IVUS examination. A quantitative IB-IVUS analysis system (VISIATLAS, Terumo, Tokyo, Japan), which measures both plaque volume and plaque composition automatically, was used for IVUS image analyses [19]. The culprit lesions were analyzed to determine two-dimensional (2D) and three-dimensional (3D) IVUS parameters. Conventional IVUS and IB-IVUS images were recorded at an interval of 1.0 mm for a length of 10 mm in each plaque at the culprit lesion (Figure 1).

### 2.6. Analysis of Acquired IVUS Images

For IVUS analysis, first, a cross-section with minimal lumen area of the culprit lesion was identified. Then, the cross-sections that were located 5 mm proximal and distal to the most diseased cross-section were identified. This process identified a 10-mm target segment for IVUS analysis, which was performed on those spaced 1.0 mm apart. The external elastic membrane (EEM) and luminal borders were manually traced in each cross-section, and EEM area (vessel area) and lumen area were automatically calculated using 2D IVUS images. Plaque area was calculated as the difference between the vessel area and the lumen area. Total atheroma area (TAA) was calculated using the summation of the plaque area in each measured image. The vessel volume, lumen volume, and total atheroma volume (TAV) were calculated via summation of those respective 2D IVUS measures. The percent atheroma area (PAA) and percent atheroma volume (PAV) were calculated as 100 × (plaque area or volume/vessel area or volume). A quantitative tissue characterization of coronary plaques was conducted using the IB-IVUS analysis system. The IB values for each plaque composition were determined according to an average power of the ultrasound backscattered signal, and plaque compositions were shown as color-coded blue (lipid), green (fibrous), yellow (dense fibrous), and red (calcification) [18,19]. Quantitative assessment of each plaque composition was automatically analyzed as follows: lipid area/volume (LA/LV), fibrous area/volume (FA/FV), dense fibrous area/volume (dense-FA/dense-FV), and calcified area/volume (CA/CV), respectively. Those percentages were calculated based on the following formula: 100 × (area or volume of each composition/plaque area or volume). IVUS analysis was conducted using an experienced physician (A.I.) who was blinded to the patient characteristics according to the criteria described in the American College of Cardiology Clinical Expert Consensus document on IVUS [27]. The intra-observer variability with representative images was 1.4% (*n* = 10).

### 2.7. Statistical Analysis

Continuous variables were presented as mean (standard deviation) or median (interquartile range), and comparisons between groups were performed using the Wilcoxon rank-sum test. Categorical variables were presented as numbers (percentages), and comparisons between groups were performed using the Chi-square test and/or Fisher’s exact test. We created multiple dichotomous variables for categorical variables. Correlations were assessed using Spearman’s correlation coefficients, and summarized in a heat map. After excluding patients with missing data (*n* = 4) and extremely high HDL-C levels (*n* = 1), we performed multivariate data analysis (MVDA) to examine the associations of CAD risk factors with coronary plaque characteristics, considering large numbers of independent variables as well as the potential for multicollinearity between them [28]. A Box-Cox transformation was applied to auto-scale the dataset before the MVDA. First, for MVDA, the correlations among variables were analyzed using principal components analysis (PCA). Principal components (PCs) were calculated based on coefficients among variables using PCA and expressed as linear transformations of the original dataset. While the score plot of PCA shows the relationships among patients according to PCs presented as axes in a two-dimensional coordinate system, the loading plot of PCA shows the relationships among variables. In addition, an orthogonal partial least squares (OPLS) regression was performed to examine the associations between the predictor variables (X variables) and the outcome variables (Y variables). The OPLS regression model contained two predictive components (predictive X-Y, P1 and P2), and one X component (orthogonal predictor variable: O1) which was independent of the outcome variables. In addition to regression coefficients, variable importance in projection (VIP) scores were calculated in the OPLS model to identify the relative importance of each independent variable. A two-sided *p* value of less than 0.05 was considered to be statistically significant unless indicated otherwise. MVDA was performed using SIMCA version 14 (MKS Umetrics AB, Umeå, Sweden). Other statistical analyses were performed using SAS version 9.4 (SAS Institute, Cary, NC, USA). 

## 3. Results

### 3.1. Patient Characteristics

Patient characteristics are summarized in Table 1. The mean age was 70 years of which 68% were male. The frequencies of hypertension and dyslipidemia were 79% and 84%, respectively. Statins were used by 97% of patients. In a single case a patient was treated with ezetimibe alone as a first-line lipid lowering therapy by the treating clinician’s choice. A history of prior PCI was found in 15 of the 38 patients (39%), and 76% of patients showed multivessel disease. Table 2 shows the degrees of cardiovascular risk factors and HDL functionality. The mean LDL-C levels were 88 mg/dL, satisfying the Japanese guidelines for the secondary prevention of cardiovascular diseases [23,24]. Despite this study cohort being a specific T2DM population, diabetic dyslipidemia characterized by elevated triglyceride (TG) and low HDL-C levels was not observed in the current study (mean TG: 149 mg/dL; mean HDL-C: 50 mg/dL, respectively). The HII did, however, indicate reduced HDL antioxidant capacity with mean levels above 1.0 [mean HII 1.10 AU (arbitrary unit)].

### 3.2. Gray-Scale and IB-IVUS Parameters at the Culprit Lesion

A total of 760 Gray-IVUS and IB-IVUS images were analyzed (Gray-scale IVUS: 380, IB-IVUS: 380, respectively). Gray-scale and IB-IVUS parameters at the culprit lesion are summarized in Table 3. The mean PAV was above 70% (73.3 ± 5.5%), and the plaque composition was predominantly lipid in this study population [3D IVUS parameters: %LV {57.1 (45.0, 66.4)}, %FV {36.5 (28.3, 44.0)}, 2D IVUS parameters: %LA {62.2 (55.3, 72.0)}, %FA {31.1 (24.7, 36.4)}]. In addition, the amount of plaque burden was smaller in female patients, although the findings were inconclusive due to our small sample size.

### 3.3. Correlations between IVUS-Derived Measures and Clinical Demographics, including HDL Functionality

**Figure 2:**
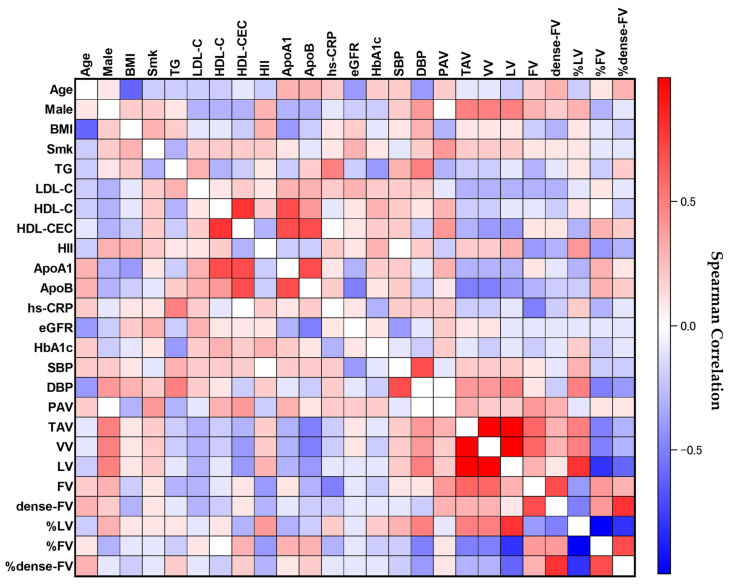
Correlations between IVUS-derived measures and clinical demographics, including HDL functionality. The heat map explains correlations between IVUS-derived measures and clinical demographics, including HDL functionality. Darker red indicates stronger positive correlations, and darker blue indicates stronger negative ones. ApoA1 = apolipoprotein A-1, ApoB = apolipoprotein B, BMI = body mass index, DBP = diastolic blood pressure, dense-FV = dense fibrous volume, eGFR = estimated glomerular filtration rate, FV = fibrous volume, HbA_1c_ = glycosylated hemoglobin, HDL-C = high-density lipoprotein cholesterol, HDL-CEC = HDL-mediated cholesterol efflux capacity, HII = HDL inflammatory index, hs-CRP = high sensitivity C-reactive protein, HT = hypertension, IVUS = intravascular ultrasound, LDL-C = low-density lipoprotein cholesterol, LV = lipid volume, PAV = percent atheroma volume, SBP = systolic blood pressure, Smk = smoking, TAV = total atheroma volume, TG = triglyceride, VV = vessel volume, %dense-FV = percentage of dense fibrous volume, %FV = percentage of fibrous volume, %LV = percentage of lipid volume.

Figure 2 shows the heat map for spearman correlations of IVUS-derived measures with patient demographics and HDL functionality. The positive and negative correlations among variables are presented as red and blue in the heat map. The color shading reflects the coefficient of correlation: the stronger the correlation is, the darker the color shading becomes. HDL-C levels were strongly correlated with HDL-CEC (r = 0.74, *p* < 0.001) positively but not with HII (*p* = 0.35). HDL-CEC was positively correlated with atheroma burden (PAA: *p* = 0.96; PAV: r = 0.34, *p* = 0.04, respectively) whereas HII was not (PAA: *p* = 0.73; PAV: *p* = 0.65, respectively).

With regard to plaque composition assessed by IB-IVUS, HDL functionality did not show significant correlations with dense fibrous and calcified compositions. In contrast, HII significantly correlated with fibrous and lipid compositions (%FA: r = −0.36, *p* = 0.03; %FV: r = −0.42, *p* = 0.01; %LA: r = 0.36, *p* = 0.03; %LV: r = 0.39, *p* = 0.02, respectively). HDL-CEC also showed significant correlations with those compositions (%FA: r = 0.44, *p* = 0.01; %FV: r = 0.31 *p* = 0.07; %LA: r = −0.38, *p* = 0.02; %LV: r = −0.28, *p* = 0.11, respectively).

### 3.4. HDL Functionality and Coronary Plaques

**Figure 3:**
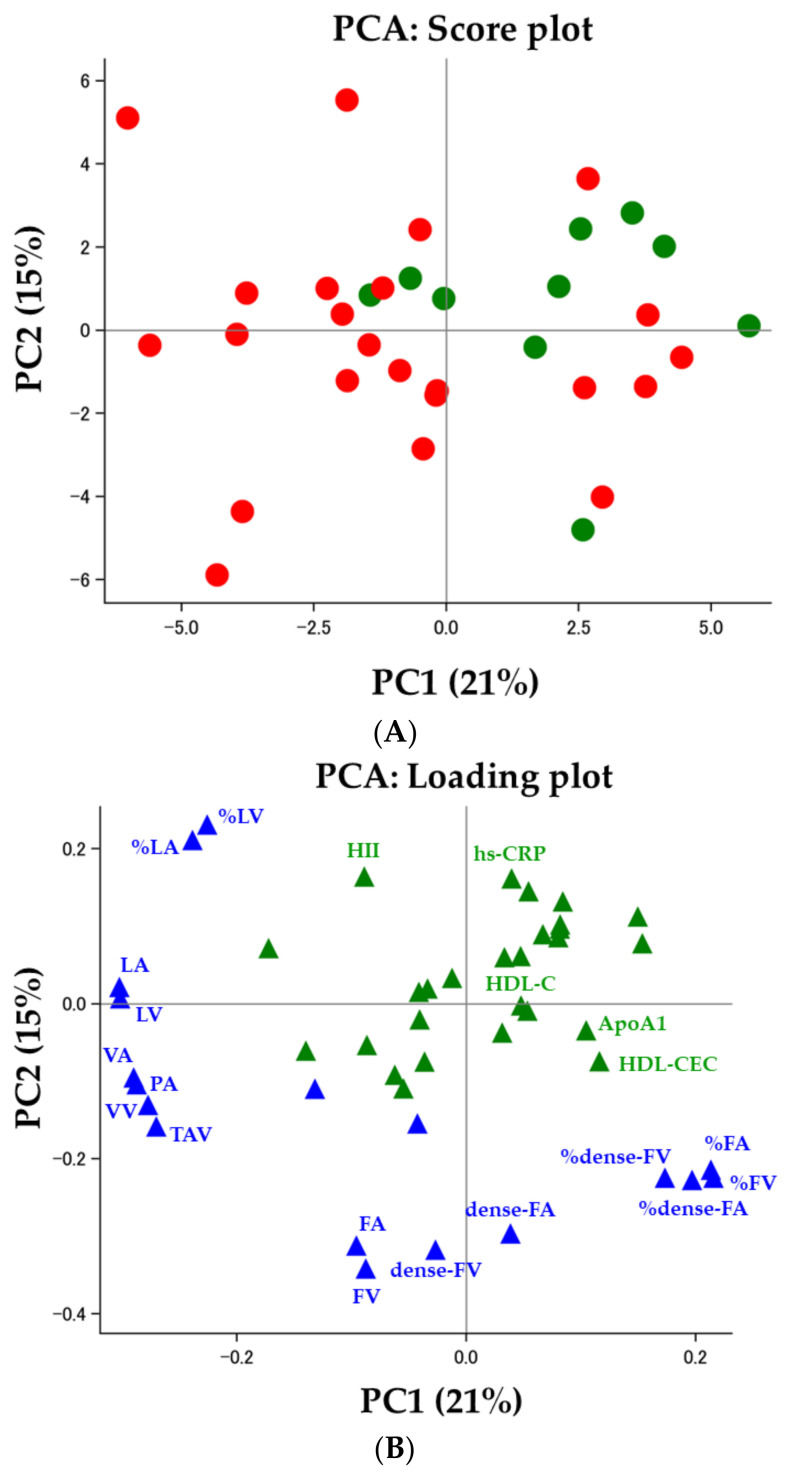
Principal Component Analysis (Score Plot and Loading Plot). (**A**) Score plot: While males were illustrated in red, females were shown in green. Females are clustered together in the higher half of PC1 and PC2, while males appear to be predominantly in the lower half of PC1 but not as clearly clustered together as females; (**B**) Loading plot: Variables are shown as follows: patient demographics and HDL-related parameters in green, and IVUS-derived measures in blue. ApoA1 = apolipoprotein A-1, dense-FA = dense fibrous area, dense-FV = dense fibrous volume, FA = fibrous area, FV = fibrous volume, HDL-C = high-density lipoprotein cholesterol, HDL-CEC = HDL-mediated cholesterol efflux capacity, HII = HDL inflammatory index, hs-CRP = high sensitivity C-reactive protein, IVUS = intravascular ultrasound, LA = lipid area, LV = lipid volume, PA = plaque area, PC = principal component, PCA = principal components analysis, TAV = total atheroma volume, VA = vessel area, VV = vessel volume, %dense-FA = percentage of dense fibrous area, %dense-FV = percentage of dense fibrous volume, %FA = percentage of fibrous area, %FV = percentage of fibrous volume, %LA = percentage of lipid area, %LV = percentage of lipid volume.

As illustrated in Figure 3 (Figure 3A: score plot, Figure 3B: loading plot), we performed PCA with forty-five variables from twenty-five demographic variables (green in Figure 3B), sixteen IVUS-derived measures (blue in Figure 3B), and four HDL-related variables (green in Figure 3B). The first two components explained 36% of the total variance as shown in the loading plot (PC1: 21%, PC2: 15%, respectively, Figure 3B). While females appear to cluster together in the higher half of PC1 and PC2, males display more variance across both PCs (male: red, female: green, Figure 3A). While PC1 was associated with vessel area, vessel volume, atheroma burden, and plaque composition (all of lipid-related measures and volume and area percentage of fibrous and dense fibrous), PC2 was associated with HII, hs-CRP, and plaque composition (volume and area of fibrous, dense fibrous, and percentage of lipid). Three-dimensional IVUS parameters showed comparable loading values to 2D IVUS parameters (Figure 3B).

**Figure 4:**
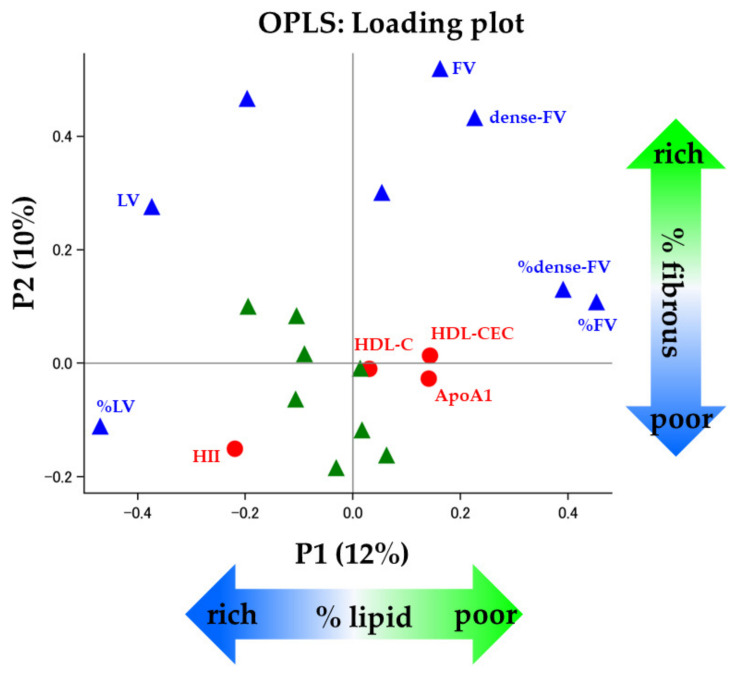
The loading plot from OPLS model. While conventional ASCVD risk factors are shown in green, HDL-related parameters are shown in red. IVUS-derived measures are shown in blue. ApoA1 = apolipoprotein A1, ASCVD = atherosclerotic cardiovascular disease, dense-FV = dense fibrous volume, FV = fibrous volume, HDL-C = high-density lipoprotein cholesterol, HDL-CEC = HDL-mediated cholesterol efflux capacity, HII = HDL inflammatory index, IVUS = intravascular ultrasound, LV = lipid volume, OPLS = orthogonal projections to latent structures, P1 = predictive component 1, P2 = predictive component 2, %dense-FV = percentage of dense fibrous volume, %FV = percentage of fibrous volume, %LV = percentage of lipid volume.

**Figure 5:**
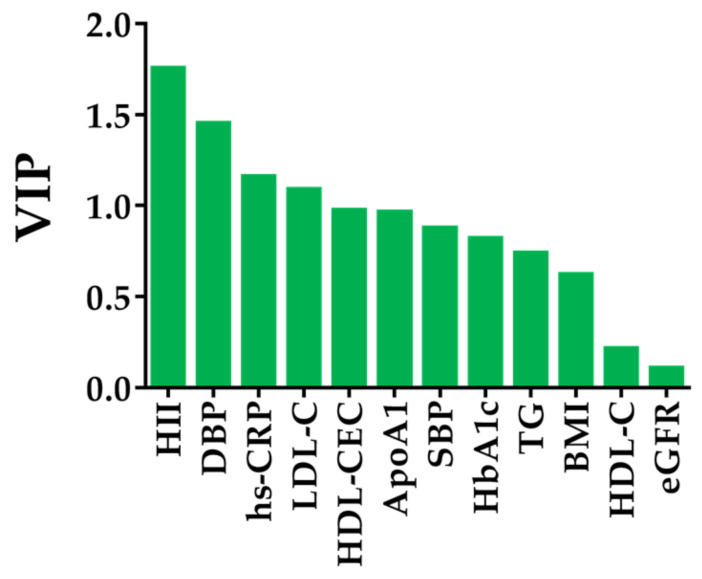
Relative Importance of the Independent Variables for Coronary Plaque Characteristics. OPLS model calculated VIP scores which reflects the relative importance of each variable for plaque characteristics. A variable with a higher VIP score indicates greater involvement with plaque characteristics. HDL antioxidant capacity showed the largest VIP score. ApoA1 = apolipoprotein A1, BMI = body mass index, DBP = diastolic blood pressure, eGFR = estimate glomerular filtration rate, HbA_1c_ = glycosylated hemoglobin, HDL-C = high-density lipoprotein cholesterol, HDL-CEC = HDL-mediated cholesterol efflux capacity, HII = HDL inflammatory index, hs-CRP = high-sensitivity C-reactive protein, LDL-C = low-density lipoprotein cholesterol, OPLS = orthogonal partial least squares, SBP = systolic blood pressure, TG = triglyceride, VIP = variable importance in projection.

In the OPLS regression model (Figure 4), the first two predictive components (P1 and P2) explained 22% of the total variance in predictor variables (P1: 12%, P2: 10%, respectively) whereas they represented 81% of those in outcome variables which were described as dependent variables in Table 4 (P1: 45%, P2: 36%, respectively). The orthogonal predictor variable (O1) explained 16% of the predictor variables. The loading values of P1 and P2 were summarized in Table 5, and Figure 4 shows those plots. In terms of the relationships with IVUS-derived measures (Table 4), P1 explained around 50% of the variance in %LV and %FV (%LV: 49%; %FV: 47%, respectively, Table 4). On the other hand, P2 explained 35% of the variance in FV (Table 4). These findings indicated that the first two predictive components represent coronary plaque compositions, reflecting coronary plaque vulnerability. In the subsequent OPLS regression models, we examined the association of independent variables with %LV and FV which showed the biggest loading value of P1 and P2, respectively. The OPLS regression demonstrated that HII was positively associated with %LV (regression coefficient (95% confidence interval): 0.34 (0.08 to 0.60), Table 6). Meanwhile HDL-CEC showed an inverse association with %LV (−0.26 (−0.49 to −0.04), Table 6). Furthermore, the VIP score of HII was the highest among the independent variables including HDL-related parameters, suggesting it had the greatest contribution to coronary plaque characteristics (VIP scores, HII: 1.75, HDL-CEC: 0.97, HDL-C: 0.21, respectively, Figure 5).

## 4. Discussion

Coronary atheroma progression despite achieving target LDL-C levels requires novel targets to improve cardiovascular outcomes. T2DM patients tend to exhibit unstable coronary plaque phenotype such as lipid-rich plaques. In the current study, we used PCA followed by OPLS model for detecting predictors of plaque characteristics given the limitation of classical regression models in managing large numbers of variables for our sample size. The detection sensitivity of the OPLS model was reported to be superior to that of classical regression models [29]. An impaired HDL-CEC in CAD patients with extremely high HDL-C levels was reported [30]. Even in regard to the antioxidative capacity of HDL, the property of HDL assessed by using the same measuring system as ours, was reported to be reduced in coronary heart disease patients with extremely high HDL-C (mean HDL-C levels: 95 mg/dL) [13]. In this study, a patient with extremely high HDL-C level (99 mg/dL) was excluded due to the effects of the outlier on the statistics (3 sigma rule). In the OPLS model, coronary plaque composition was associated with both HDL antioxidant capacity and HDL-CEC in T2DM patients who were maintained in accordance with the local national guidelines of lipids, blood pressure, and T2DM. The VIP scores of HDL functionality were much stronger than that of HDL-C level, indicating great importance of HDL functionality for coronary plaque characteristics. Above all, the highest VIP value of HII highlighted the major implication of HDL antioxidant capacity to coronary plaque characteristics. Furthermore, the HDL antioxidant capacity was significantly associated with %LV, indicating involvements of the HDL property on plaque vulnerability reflected by plaque composition. To our knowledge, this is the first report of relationships between HDL functionality and coronary plaque composition assessed using IB-IVUS. Our findings underscore the potential importance of HDL functionality in plaque stabilization of T2DM patients with CAD.

In this study, patient and plaque characteristics were stratified by gender given the established gender difference in HDL-C levels. Although our sample size is too small to statistically detect gender differences of plaque composition, our observed associations between gender and plaque characteristics were consistent with previous studies [31]. In addition, we performed OPLS analysis to further compensate for the discordance between small sample size and larger numbers of independent variables. Our OPLS models demonstrated that HDL antioxidant capacity exerted the greatest contributions to coronary plaque characteristics in T2DM patients with CAD. Increased oxidative stress in T2DM has been reported to contribute to a more vulnerable plaque phenotype [32,33,34,35]. Excessive oxidative stress increases oxidized low-density lipoprotein (LDL) and other lipid oxidation products. While HDL exerts its anti-inflammatory properties on protection of LDL against oxidation [36,37], HDL loses its atheroprotective characteristics in inflammatory conditions like ACS and T2DM [16,17]. In fact, the mean level of HII in this study was considered to be pro-oxidant according to previous studies using HII [14,38]. Activated inflammasomes, induced by increased oxidative stress, degrade collagen fibers and reduces the production of vascular smooth muscle cells (VSMCs), which is protective against the destabilization of atherosclerotic plaques [39]. In response, HDL antioxidant capacity inhibits the accumulation of lipid hydroperoxides and prevents the following inflammatory responses [36,37]. Furthermore, suppressed inflammatory responses in VSMCs were also verified by pretreatment with HDL [40]. These mechanisms could support the observations between greater HDL antioxidant capacity and larger fibrous tissues in the current study. In addition, HDL-CEC played an important role in reducing lipid composition of coronary plaques in this study. In particular, the cholesterol efflux has been frequently proposed to be inversely associated with ASCVD risks, independent of HDL-C levels [11,41]. These mechanisms could explain the association of HDL-CEC with lipid composition of coronary plaques in the current study.

In this study, HDL-C levels were not associated with plaque composition. The heterogeneity of HDL particle which plays a role in its atheroprotective properties could corroborate our insignificant relationships. Namely, HDL-C levels do not reflect the capacity of the functional aspect of the HDL particle. This observation may link with the aforementioned associations between HDL functionality and clinical outcomes, independent of HDL-C levels. Our observations using IB-IVUS suggest that favorable HDL functionality may stabilize coronary plaques by modifying plaque composition in T2DM patients even under optimal medical therapy. The plaque phenotype was reported to be independently associated with future cardiovascular events by a previous study using IB-IVUS [21]. In addition, our study participants were deemed to present with high-risk plaque phenotypes estimated by their PAV [42]. On the other hand, the YELLOW II Study using optical coherence tomography (OCT) revealed that a beneficial increase in fibrous cap thickness was significantly associated with an improved HDL-CEC, although the prevalence of concomitant T2DM and baseline PAV were mild [43]. Hence, further prospective studies using OCT will be needed to elucidate the relationship between HDL functionality and coronary plaque microstructures in T2DM patients. Furthermore, the impact of HDL antioxidant capacity on changes in coronary plaque composition with serial IB-IVUS images should be examined over a longer period. The associations would be of considerable interest given poor outcomes in ACS patients with less HDL antioxidant capacity [38]. Some interventions in increasing HDL-CEC have been reported whereas little is known about an effective strategy to improve the antioxidant property assessed as HII, reflecting the net actions of HDL components [44,45,46]. Several studies reported that statin and apoA1 mimetic peptide L-4F improved this ability of HDL [13,16]. Although some apoA-I-based approaches failed to show their clinical benefits on cardiovascular events [47,48], a potential HDL infusion therapy CSL112, which utilizes human-plasma-derived apoA-I, is currently being assessed in the ongoing AEGIS-II trial [49]. CSL112 has a dramatically enhancing effect on HDL-CEC compared to previous HDL infusions [50]. The impact of these findings on therapeutic interventions targeting HDL functionality remains to be determined. In addition, HDL-bound microRNAs (miRNAs) is known to target cholesterol transporters and regulate RCT [51]. Given the contributions of multiple HDL-bound miRNAs on the development of plaque burden [52], future coronary imaging studies on HDL-miRNAs and HDL functionality are definitely intriguing.

An emerging novel drug, bempedoic acid, targets adenosine triphosphate-citrate lyase (ACLY). Bempedoic acid reduces acetyl-CoA and cholesterol synthesis by inhibiting ACLY, resulting in an increased number of LDLRs and reduced plasma cholesterol levels. ACLY is relevant to the expression of the cholesterol efflux genes ABCG5/8, resulting in the mediation of hepatic cholesterol efflux. Recently, an in vitro and in vivo study reported increasing cholesterol efflux from using the ACLY inhibitor [53]. This finding may contribute to a promising future of bempedoic acid for preventing ASCVD, in addition to the well-known effect of reducing LDL-C and CRP levels in the CLEAR outcomes trial [54].

We acknowledge several limitations. This study was conducted in a single center with a small sample size. The association of HDL functionality with measurements of IVUS and IB-IVUS parameters were evaluated in a cross-sectional study design. Further larger multicenter, longitudinal studies with serial IVUS examination and blood samples at each follow-up are warranted to confirm our findings. One potential source of selection bias in this study is the exclusion criteria. The potential impact of the exclusion criteria on our findings is uncertain.

## 5. Conclusions

HDL antioxidant capacity and HDL-CEC were associated with coronary plaque composition, which have been considered to be important factors for plaque vulnerability. HDL functionality may be still a potential therapeutic target to reduce cardiovascular events in T2DM patients with CAD, although recent HDL-C and HDL targeted therapies, including HDL infusions, failed to show their benefits.

## Figures and Tables

**Table 1:** Patient Characteristics.

Variables	Overall (*n* = 38)	Male (*n* = 26)	Female (*n* = 12)	*p* Value
Age, years	70 ± 9	70 ± 8	70 ± 11	0.90
BMI, kg/m^2^	25 ± 4	25 ± 4	24 ± 5	0.50
Smoking, *n* (%)	5 (13)	4 (15)	1 (8)	0.55
Hypertension, *n* (%)	30 (79)	21 (81)	9 (75)	0.69
Dyslipidemia, *n* (%)	32 (84)	22 (85)	10 (83)	0.92
Hyperuricemia, *n* (%)	6 (16)	6 (23)	0 (0)	0.07
CKD, *n* (%)	20 (53)	15 (58)	5 (42)	0.36
Prior MI, *n* (%)	8 (21)	5 (19)	3 (25)	0.69
Prior PCI, *n* (%)	15 (39)	9 (35)	6 (50)	0.37
Prior CABG, *n* (%)	1 (3)	1 (4)	0 (0)	0.49
Number of diseased vessel, *n* (%) 1/2/3	18/11/9 (47/29/24)	14/8/4 (54/31/15)	4/3/5 (33/25/42)	0.20
Target vessel, *n* (%) LAD/LCx/RCA/LMT	14/5/18/1 (37/13/47/3)	8/3/14/1 (31/12/54/4)	6/2/4/0 (50/17/33)	0.54
Stent, *n* (%) DES/BMS	37/1 (97/3)	25/1 (96/4)	12/0 (100/0)	0.49
Medications, *n* (%)				
Statin	37 (97)	25 (96)	12 (100)	0.49
Ezetimibe	3 (8)	3 (12)	0 (0)	0.22
EPA	7 (18)	5 (19)	2 (17)	0.85
ARB	25 (66)	18 (69)	7 (58)	0.51
ACE-I	2 (5)	1 (4)	1 (8)	0.56
CCB	26 (68)	16 (62)	10 (83)	0.18
β-blocker	3 (8)	3 (12)	0 (0)	0.22
Aspirin	38 (100)	26 (100)	12 (100)	1.00
Thienopyridine	38 (100)	26 (100)	12 (100)	1.00

Data are presented as mean (standard deviation) or numbers (percentage). ACE-I = angiotensin converting enzyme inhibitor, ARB = angiotensin receptor blocker, BMI = body mass index, BMS = bare metal stent, CABG = coronary artery bypass grafting, CCB = calcium channel blocker, CKD = chronic kidney disease, DES = drug-eluting stent, EPA = eicosapentaenoic acid, GI = glucosidase inhibitor, LAD = left anterior descending artery, LCx = left circumflex artery, LMT = left main trunk, MI = myocardial infarction, PCI = percutaneous coronary intervention, RCA = right coronary artery.

**Table 2:** Cardiovascular Risk Factors and HDL Functionality.

Variables	Overall (*n* = 38)	Male (*n* = 26)	Female (*n* = 12)	*p* Value
Lipids				
TG, mg/dL	149 ± 76	152 ± 75	144 ± 82	0.69
LDL-C, mg/dL	88 ± 24	84 ± 26	96 ± 20	0.12
sd-LDL-C, mg/dL	32 ± 15	32 ± 15	34 ± 16	0.64
HDL-C, mg/dL	50 ± 14	47 ± 11	55 ± 18	0.18
HDL functionality				
HDL-CEC, %	14.2 ± 1.4	13.9 ± 1.3	14.7 ± 1.5	0.12
HII, AU	1.10 ± 0.20	1.12 ± 0.19	1.05 ± 0.24	0.18
Apolipoproteins				
ApoA1, mg/dL	94 ± 22	89 ± 21	103 ± 22	0.10
ApoB, mg/dL	22 ± 8	20 ± 6	26 ± 10	0.10
ApoC2, mg/dL	4.8 ± 1.8	4.7 ± 1.7	4.9 ± 2.0	0.63
ApoC3, mg/dL	12.9 ± 3.3	12.8 ± 3.1	13.2 ± 3.7	0.55
Free fatty acids				
LnA, µg/mL	34 ± 11	33 ± 12	37 ± 9	0.18
AA, µg/mL	173 ± 31	165 ± 28	190 ± 33	0.025
EPA, µg/mL	80 ± 57	82 ± 53	77 ± 68	0.15
EPA/AA	0.50 ± 0.42	0.53 ± 0.42	0.43 ± 0.43	0.06
DHA, µg/mL	125 ± 35	125 ± 37	127 ± 34	0.76
Others				
hs-CRP, mg/dL	0.13 ± 0.20	0.13 ± 0.23	0.11 ± 0.10	0.75
eGFR, mL/min/1.73 m^2^	61 ± 15	59 ± 14	64 ± 17	0.43
UA, mg/dL	5.2 ± 1.4	5.6 ± 1.4	4.4 ± 1.1	0.015
HbA_1c_, %	7.0 ± 1.0	7.0 ± 1.1	7.1 ± 0.8	0.38
FBS, mg/dL	103 ± 23	106 ± 21	98 ± 26	0.20
SBP, mmHg	129 ± 17	131 ± 19	124 ± 11	0.43
DBP, mmHg	69 ± 12	72 ± 12	64 ± 8	0.038
LVEF, %	66 ± 9	64 ± 10	69 ± 8	0.18

Data are presented as mean (standard deviation). AA = arachidonic acid, ApoA1 = apolipoprotein A-1, ApoB = apolipoprotein B, ApoC2 = apolipoprotein C2, ApoC3 = apolipoprotein C3, AU = arbitrary unit, DBP = diastolic blood pressure, DHA = docosahexaenoic acid, eGFR = estimated glomerular filtration rate, EPA = eicosapentaenoic acid, EPA/AA = the ratio of EPA to AA, FBS = fasting blood sugar, HbA_1c_ = glycosylated hemoglobin, HDL-C = high-density lipoprotein cholesterol, HDL-CEC = HDL-mediated cholesterol efflux capacity, hs-CRP = high-sensitivity C-reactive protein, LDL-C = low-density lipoprotein cholesterol, LnA = linoleic acid, LVEF = left ventricular ejection fraction, SBP = systolic blood pressure, sd-LDL-C = small-dense LDL-C, TG = triglyceride, UA = uric acid.

**Table 3:** Gray-scale IVUS and IB-IVUS-derived measures.

Variables	Overall (*n* = 38)	Male (*n* = 26)	Female (*n* = 12)	*p* Value
Gray-scale IVUS parameters				
3D parameters				
PAV, %	73.3 ± 5.5	73.4 ± 5.5	72.9 ± 5.8	1.00
TAV, m^3^	87.4 (67.9 to 102.6)	91.6 (82.3 to 113.0)	72.8 (54.2 to 84.9)	0.005
Vessel volume, m^3^	117.8 (87.7 to 137.9)	125.3 (108.8 to 152.5)	94.0 (77.9 to 110.8)	0.005
Lumen volume, m^3^	27.4 (23.8 to 39.3)	32.3 (24.8 to 41.3)	24.5 (20.9 to 27.2)	0.01
2D parameters				
PAA, %	82.8 ± 5.5	83.4 ± 5.9	81.6 ± 4.4	0.23
TAA, m^2^	10.4 (7.5 to 13.3)	11.5 (9.0 to 15.3)	7.9 (6.8 to 9.4)	0.01
Vessel area, m^2^	12.1 (9.3 to 15.5)	14.0 (10.8 to 16.9)	9.5 (8.8 to 11.6)	0.01
Lumen area, m^2^	1.8 (1.5 to 2.5)	1.9 (1.6 to 2.5)	1.6 (1.3 to 2.3)	0.16
IB-IVUS parameters				
3D parameters				
Plaque components				
LV, m^3^	48.8 (31.8 to 62.6)	58.9 (43.2 to 77.5)	37.7 (29.4 to 46.9)	0.01
FV, m^3^	28.8 (23.6 to 35.3)	30.2 (26.4 to 37.0)	25.3 (19.9 to 33.0)	0.15
dense-FV, m^3^	3.1 (2.2 to 5.5)	3.6 (2.3 to 6.1)	2.6 (1.8 to 4.2)	0.26
CV, m^3^	0.9 (0.4 to 1.8)	0.9 (0.4 to 1.8)	0.7 (0.3 to 1.9)	0.68
Plaque composition				
%LV	57.1 (45.0 to 66.4)	62.0 (45.0 to 68.2)	56.2 (47.0 to 58.3)	0.13
%FV	36.5 (28.3 to 44.0)	33.8 (27.1 to 42.5)	37.6 (31.6 to 51.0)	0.24
%dense-FV	4.4 (2.5 to 5.8)	4.2 (2.4 to 5.8)	4.7 (2.6 to 5.8)	0.63
%CV	1.2 (0.4 to 1.9)	1.2 (0.4 to 1.4)	1.3 (0.5 to 2.9)	0.58
2D parameters				
Plaque components				
LA, m^2^	6.7 (4.2 to 8.8)	7.9 (6.4 to 10.4)	4.3 (4.0 to 5.4)	0.01
FA, m^2^	2.7 (2.3 to 4.0)	3.1 (2.3 to 4.0)	2.4 (2.3 to 2.6)	0.16
dense-FA, m^2^	0.4 (0.2 to 0.5)	0.4 (0.3 to 0.6)	0.2 (0.2 to 0.4)	0.17
CA, m^2^	0.1 (0.0 to 0.2)	0.1 (0.0 to 0.2)	0.1 (0.0 to 0.2)	0.63
Plaque composition				
%LA	62.2 (55.3 to 72.0)	66.9 (55.3 to 75.7)	59.4 (55.3 to 62.8)	0.12
%FA	31.1 (24.7 to 36.4)	30.2 (22.1 to 35.7)	32.7 (29.7 to 40.3)	0.14
%dense-FA	3.5 (2.1 to 6.0)	3.1 (2.1 to 6.0)	3.9 (2.1 to 5.4)	0.94
%CA	0.8 (0.2 to 2.1)	0.7 (0.2 to 2.1)	0.9 (0.3 to 2.0)	0.79

Values are mean (standard deviation) or median (interquartile range). CA = calcified area, CV = calcified volume, dense-FA = dense fibrous area, dense-FV = dense fibrous volume, FA = fibrous area, FV = fibrous volume, IB-IVUS = integrated backscatter intravascular ultrasound, IVUS = intravascular ultrasound, LA = lipid area, LV = lipid volume, PAA = percent atheroma area, PAV = percent atheroma volume, TAA = total atheroma area, TAV = total atheroma volume, %CA = percentage of calcified area, %CV = percentage of calcified volume, %dense-FA = percentage of dense fibrous area, %dense-FV = percentage of dense fibrous volume, %FA = percentage of fibrous area, %FV = percentage of fibrous volume, %LA = percentage of lipid area, %LV = percentage of lipid volume.

**Table 4:** Variations in the IVUS-derived measures of the OPLS model.

Dependent Variable	Variations by P1	Variations by P2
PAV	<0.001	0.03
TAV	0.15	0.24
LV	0.46	0.08
FV	0.03	0.35
dense-FV	0.04	0.10
%LV	0.49	0.004
%FV	0.47	0.03
%dense-FV	0.18	0.001

dense-FV = dense fibrous volume, FV = fibrous volume, IVUS = intravascular ultrasound, LV = lipid volume, OPLS = orthogonal partial least squares, PAV = percent atheroma volume, P1 = predictive component 1, P2 = predictive component 2, TAV = total atheroma volume, %dense-FV = percentage of dense fibrous volume, %FV = percentage of fibrous volume, %LV = percentage of lipid volume.

**Table 5:** Loading Values of the 1st and 2nd Predictive Components in the 2 + 1 OPLS Model.

	Loading Value of P1	Loading Value of P2
X variables		
BMI	−0.09	0.02
TG	0.02	−0.12
LDL-C	0.06	−0.16
HDL-C	0.03	−0.01
HDL-CEC	0.14	0.01
HII	−0.22	−0.15
ApoA1	0.14	−0.03
hs-CRP	−0.03	−0.18
eGFR	0.01	−0.01
HbA_1c_	−0.11	−0.06
SBP	−0.10	0.08
DBP	−0.19	0.10
Y variables		
PAV	0.05	0.30
TAV	−0.20	0.47
LV	−0.37	0.28
FV	0.16	0.52
dense-FV	0.23	0.43
%LV	−0.47	−0.11
%FV	0.45	0.11
%dense-FV	0.39	0.13

ApoA1 = apolipoprotein A-1, BMI = body mass index, DBP = diastolic blood pressure, dense-FV = dense fibrous volume, eGFR = estimate glomerular filtration rate, FV = fibrous volume, HbA_1c_ = glycosylated hemoglobin, HDL-C = high-density lipoprotein cholesterol, hs-CRP = high-sensitivity C-reactive protein, LDL-C = low-density lipoprotein cholesterol, LV = lipid volume, OPLS, = orthogonal partial least squares, PAV = percent atheroma volume, P1 = predictive component 1, P2 = predictive component 2, SBP = systolic blood pressure, TAV = total atheroma volume, TG = triglyceride, %dense-FV = percentage of dense fibrous volume, %FV = percentage of fibrous volume, %LV = percentage of lipid volume.

**Table 6:** OPLS Regression Models of Scaled and Centered Variables for %LV and FV.

Variables	%LV		FV	
	Coefficient	95% CI	Coefficient	95% CI
HII	0.34	(0.08, 0.60)	−0.25	(−0.68, 0.17)
DBP	0.28	(0.01, 0.54)	0.20	(−0.37, 0.77)
hs-CRP	0.06	(−0.13, 0.25)	−0.25	(−0.55, 0.04)
LDL-C	−0.17	(−0.47, 0.14)	−0.11	(−0.66, 0.43)
HDL-CEC	−0.26	(−0.49, −0.04)	0.15	(−0.31, 0.60)
ApoA1	−0.20	(−0.44, 0.05)	0.06	(−0.25, 0.37)
SBP	0.04	(−0.43, 0.52)	0.09	(−0.25, 0.43)
HbA_1c_	0.24	(−0.20, 0.68)	−0.20	(−0.57, 0.17)
TG	−0.03	(−0.25, 0.19)	−0.19	(−0.62, 0.25)
BMI	−0.01	(−0.26, 0.23)	−0.01	(−0.42, 0.40)
HDL-C	0.09	(−0.16, 0.33)	−0.12	(−0.48, 0.24)
eGFR	−0.07	(−0.38, 0.24)	0.10	(−0.46, 0.66)

ApoA1 = apolipoprotein A1, BMI = body mass index, CI = confidence interval, DBP = diastolic blood pressure, eGFR = estimate glomerular filtration rate, FV = fibrous volume, HbA_1c_ = glycosylated hemoglobin, HDL-C = high-density lipoprotein cholesterol, HDL-CEC = HDL-mediated cholesterol efflux capacity, HII = HDL inflammatory index, hs-CRP = high-sensitivity C-reactive protein, LDL-C = low-density lipoprotein cholesterol, OPLS = orthogonal partial least squares, SBP = systolic blood pressure, TG = triglyceride, %LV = percentage of lipid volume.

## Data Availability

The data presented in this study are available on request from the corresponding author.
